# Extracellular Vesicles-Encapsulated Yeast Prions and What They Can Tell Us about the Physical Nature of Propagons

**DOI:** 10.3390/ijms22010090

**Published:** 2020-12-23

**Authors:** Mehdi Kabani

**Affiliations:** Molecular Imaging Research Center (MIRCen), Laboratoire des Maladies Neurodégénératives (UMR9199), Université Paris-Saclay, CEA, CNRS, F-92265 Fontenay-aux-Roses, France; Mehdi.Kabani@cnrs.fr

**Keywords:** yeast, prion, *SUP35*, [*PSI*^+^], extracellular vesicles, exosomes, protein aggregation, protein quality control

## Abstract

The yeast *Saccharomyces cerevisiae* hosts an ensemble of protein-based heritable traits, most of which result from the conversion of structurally and functionally diverse cytoplasmic proteins into prion forms. Among these, [*PSI*^+^], [*URE3*] and [*PIN*^+^] are the most well-documented prions and arise from the assembly of Sup35p, Ure2p and Rnq1p, respectively, into insoluble fibrillar assemblies. Yeast prions propagate by molecular chaperone-mediated fragmentation of these aggregates, which generates small self-templating seeds, or propagons. The exact molecular nature of propagons and how they are faithfully transmitted from mother to daughter cells despite spatial protein quality control are not fully understood. In [*PSI*^+^] cells, Sup35p forms detergent-resistant assemblies detectable on agarose gels under semi-denaturant conditions and cytosolic fluorescent puncta when the protein is fused to green fluorescent protein (GFP); yet, these macroscopic manifestations of [*PSI*^+^] do not fully correlate with the infectivity measured during growth by the mean of protein infection assays. We also discovered that significant amounts of infectious Sup35p particles are exported via extracellular (EV) and periplasmic (PV) vesicles in a growth phase and glucose-dependent manner. In the present review, I discuss how these vesicles may be a source of actual propagons and a suitable vehicle for their transmission to the bud.

## 1. Introduction

**Figure 1:**
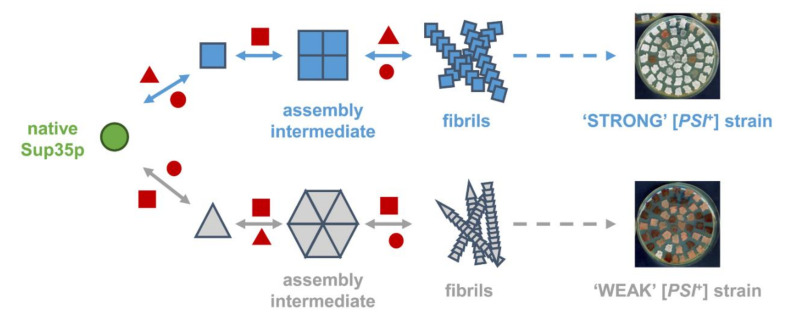
Sup35p assembles into structurally distinct fibrils leading to phenotypically distinct [*PSI*^+^] strains. Native Sup35p (green circle) can switch to alternate conformations (blue rectangle, grey triangle) that follow different assembly pathways to form structurally distinct fibrillar assemblies. The equilibrium between soluble monomers, oligomers and larger aggregated and fibrillar species is governed by protein folding and protein quality control systems (red squares, triangles and circles), comprising, for instance, molecular chaperones, the 26S proteasome or members of the endosomal–autophagic pathway. Structurally diverse Sup35p assemblies drive the formation of phenotypically distinct [*PSI*^+^] strains. These are scored on adenine-limiting plates using a genetic tool based upon the extent of nonsense suppression of the *ade1–14* allele: [*PSI*^+^] colonies appear white or pink and are prototrophic for adenine, whereas [*psi*^-^] prion-free cells form red colonies and are auxotrophic for adenine [41].

Prions are self-replicating misfolded conformations of proteins that cause dominant and heritable epigenetic traits in mammals, filamentous fungi and yeasts [1,2,3,4,5]. Over the past few decades, many prions have been described in the yeast *Saccharomyces cerevisiae* (Table 1). [*PSI*^+^], [*URE3*] and [*PIN*^+^], which correspond to the prion forms of Sup35p, Ure2p and Rnq1p, respectively, are the most extensively studied (reviewed in [6,7,8,9,10]). A common feature shared by most of these prion proteins is their ability to assemble into highly ordered self-replicating β-rich aggregates, most often of an amyloid nature [6,7,10]. Prion assembly into fibrils occurs by nucleated polymerization. This process can be reproduced in vitro using purified recombinant proteins [7,8,11,12,13,14,15]. Newly synthesized monomers switch to an assembly-competent conformation and are recruited at the ends of pre-existing homologous aggregates (Figure 1) [7,8]. The large conformational landscape of prion proteins leads to the formation of distinct strains with different seeding propensities and prion phenotypes (Figure 1) [16,17,18,19]. These high-molecular prion assemblies are dynamic and are engaged and remodeled by molecular chaperones (e.g., Hsp104, Hsp70, Hsp40), proteolytic systems and other components of the protein quality control machinery (Figure 1) (reviewed in [7,8,10,20]).

**Figure 2:**
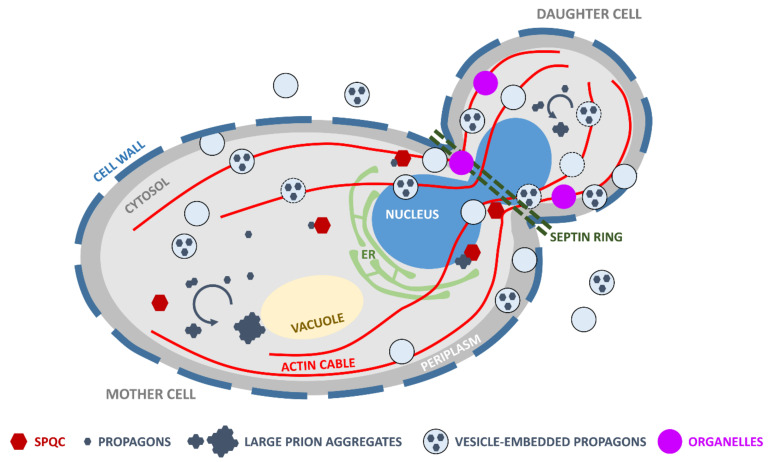
Hypothetical model of vesicle-mediated transmission of prion particles in the context of bud formation and spatial quality control. Yeast prions self-replicate by the conversion of newly synthesized soluble monomers to a prion conformation and their subsequent incorporation within high-molecular-weight prion assemblies (solid blue arrow). Unidentified prion species or “propagons” (hexagons) are cytoplasmically transmitted to the daughter cell where they initiate a new round of prion self-replication (solid blue arrow). Cytosolic prion particles are engaged by components of the spatial protein quality control (SPQC; red hexagons), such as molecular chaperones and proteolytic machineries, which ensure that most aggregates are not transmitted to the daughter cells. In addition, crowding at the mother–daughter cells junction, due to the presence of the septin ring (green dashed lines), nucleus, actin cables, vesicles and organelles (e.g., mitochondria, endoplasmic reticulum (ER); purple circles) may prevent effective transmission of “naked” propagons. Yeast prions are packaged within vesicles that are exported across the cell wall (depicted as a thick dashed line to highlight its dynamic nature) and to the extracellular medium. Vertical transmission of yeast prions could be mediated by propagons packaged within intracellular vesicles. These vesicles may be transported on actin cables to the bud where their content would be released in the cytosol to initiate a new cycle of prion self-replication. Periplasmic vesicles could also mediate the transport of propagons by diffusion in the periplasm and reinternalization in the bud (ER: endoplasmic reticulum; SPQC: spatial protein quality control). In both cases, vesicle-encapsulated propagons may escape SPQC, resulting in efficient and faithful prion transmission [6,43].

The faithful transmission of prions from mother cells to daughter cells or mating partners is mediated by cytosolic diffusible particles named propagons [42] (Figure 2). The exact molecular nature of propagons is currently not known, as prion aggregates are heterogeneous in size and composition in vivo [6,9,43].

[*PSI*^+^] stands out among other yeast prions as it is by far the most studied and best characterized prion at the genetic, cellular, biochemical and structural levels [7,43]. We showed that the size and infectivity of Sup35p prion particles changes dramatically during growth, depending on the metabolic status of the cells [44]. We previously reported that infectious Sup35p prion particles are packaged within vesicles with a diameter ranging from ~30 to 100 nm and exported in the extracellular space [6,43,45]. Extracellular vesicles (EV) are secreted by most prokaryotic, archaeal and eukaryotic cells, even those protected by a cell wall, such as yeasts, fungi or Gram-positive bacteria [6,43,46]. In glucose-limiting conditions, surprisingly high amounts of infectious Sup35p prion particles are exported into the periplasm via periplasmic vesicles (PV) [43,47]. Such behavior was described for gluconeogenic enzymes, which are secreted into the periplasm via vacuolar import and degradation (VID) vesicles, and then internalized and degraded in the vacuole upon glucose replenishing [48,49,50]. These vesicles were shown to originate from intracellular vesicle clusters (IVC), organelles that were recently proposed to synthesize EV-associated cargo proteins in yeast [48,51]. The packaging of Sup35p within vesicles may occur at IVC in actively dividing cells, although we did not observe Sup35p degradation upon feeding glucose-starved cells with fresh glucose [47]. PV and EV differ in terms of size and protein cargo, and their secretion is differentially regulated by glucose availability [47].

In the present review, I discuss the issues that I believe remain unexplained by current models of [*PSI*^+^] propagation and present my views about how vesicle-encapsulated prion particles may not only shed light on the molecular nature of propagons but also provide plausible answers to those issues.

## 2. Questions Raised by Current [*PSI*^+^] Prion Propagation Models

Most laboratory [*PSI*^+^] prion strains were selected for their remarkably high mitotic stabilities and form phenotypically homogeneous colonies [3,18,52,53,54]. In our hands, mitotically stable [*PSI*^+^] variants are not detrimental to their recipient cells, as these grow at the same rate as isogenic [*psi*^-^] counterparts [53]. Furthermore, [*PSI*^+^] cells exhibit a prolonged chronological lifespan, which could be due to higher Sup35p levels persisting in postdiauxic shift cells [44,53,55]. Prion loss can however occur at varying yet very low frequencies, depending on the genetic background and the prion variant, resulting in the appearance of rare sectored colonies or prion-free cells in liquid cultures [3,18,52,53,54].

Both experimental and mathematical modeling approaches defined important propagon characteristics [9]. The most sophisticated of these models rightfully take into account the respective size, growth rate and age of mother and daughter cells [9,52,54]. Propagons must reach a minimal critical size of ~4–30 Sup35p molecules for proper prion maintenance and propagation [52,56,57]. Yeast cells contain ~100–1000 propagons, and mother cells contain more aggregates than daughters [52,54,58]. The partition of propagons during cell division is passive and occurs via the distribution of cytoplasm [54].

Bud formation and cell division are highly regulated processes [59,60]. A double ring of septin is assembled to separate the emerging bud from the mother cell, and the division site is crowded with macromolecules, such as the dividing nucleus, organelles (e.g., mitochondria, endoplasmic reticulum, peroxisomes), actin cables and vesicles [61,62] (Figure 2). Furthermore, cell division is asymmetric: mother cells retain most aging determinants, including misfolded and aggregated proteins, oxidatively damaged organelles (e.g., mitochondria, vacuole) or extrachromosomal rDNA circles; in turn, the daughter is “rejuvenated” and essentially free of aging determinants that might reduce its lifespan [63]. Spatial quality control mechanisms and antiprion systems ensure that protein aggregates are sequestered within protein inclusions (e.g., insoluble protein deposit (IPOD), juxta nuclear quality control compartment (JUNQ)) or cleared by proteolytic machineries (e.g., proteasome, autophagy) in the mother cell [64,65].

Hence, that an individual yeast cell contains a defined number of propagons in equilibrium with both monomers and larger aggregates, some of which are passively transmitted to the emerging bud, does not quite add up with these antiaging protecting mechanisms [10,64,65,66]. Because propagons contain the structural information required to propagate the prion conformation, they should be recognized as misfolded abnormal species by antiprion systems and either cleared or addressed to protein inclusion sites [10,64,65,66]. Due to their high numbers and small sizes, propagons may escape quality control mechanisms and diffuse through the mother–bud junction despite crowding. Because one prion particle was deemed sufficient to allow prion propagation [58], such a transmission mechanism would be sufficient to allow prion maintenance during multiple cell divisions. Nevertheless, because protein quality control mechanisms are very efficient, especially in young cells, we would expect higher [*PSI*^+^]-loss frequencies than the ones we previously observed [53]. It is noteworthy that [*PSI*^+^], [*URE3*] and [*RNQ1*] variants with low mitotic stabilities were described [18,67,68,69,70]. Hence, I speculate the existence of alternative propagon transmission pathways that could strengthen the stability of some prion variants over hundreds of generations (see below) [6,43].

## 3. What We Can Learn about the Molecular Nature of Propagons Using EV

**Figure 3:**
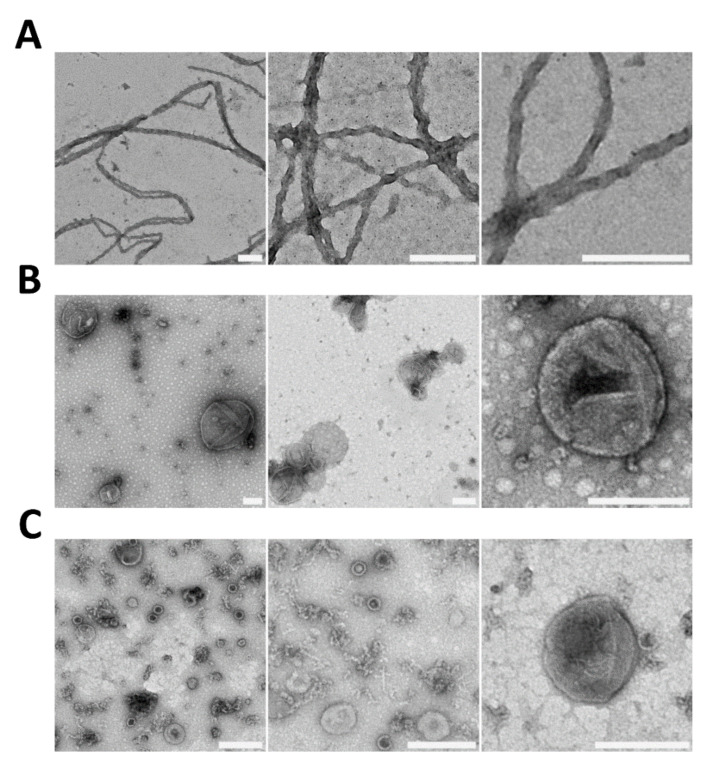
Electron micrographs of negatively stained (**A**) recombinant full-length Sup35p fibrils assembled in vitro, (**B**) extracellular vesicles (EV) purified from yeast culture medium and (**C**) periplasmic vesicles (PV) extracted from the yeast periplasm (scale bar: 200 nm) [12,45,47].

Purified recombinant full-length Sup35p assembles into amyloid fibrils [11,12,71,72,73]. In our hands, using negative staining electron microscopy (EM), Sup35p fibrils appear to be ~25 nm wide, up to several micrometers long, rigid and twisted, with a high tendency to bundle (Figure 3) [12]. Using negative staining, cryo-EM and scanning transmission EM (STEM), Steven and colleagues reported that full-length Sup35p fibrils show a thin ~8 nm wide backbone surrounded by a diffuse ~65 nm wide cloud of globular C-domains [73]. In agreement with a β-rich amyloid structure, STEM mass-per-unit-length data suggested that the fibril core consists of 1 subunit per 4.7 Å [73]. Given that the size of yeast cells is ~5–10 µm, it is unlikely that Sup35p assembles into micrometer-long fibrils within [*PSI^+^*] cells. The only evidence for the presence of Sup35p fibrils resembling those seen in vitro within cells was obtained using overexpressed tagged versions of the prion domain of Sup35p (Sup35NM) [74]. These constructs formed large dot-like and ring-like structures packed with aligned fibrillar bundles, which were ~25 nm wide with a center-to-center spacing of ~34 nm and several micrometers or ~100 nm long, respectively [74]. Transient ring, dot and thread-like structures were described by others in the process of de novo prion formation upon overexpression of GFP-tagged Sup35NM [75,76,77]. However, there is no evidence that full-length Sup35p forms such large structures in its natural context. Indeed, we and others reported that [*PSI*^+^] cells expressing a fully functional full-length Sup35GFP from the *SUP35* genomic locus show a speckled fluorescence pattern that differs from the large foci obtained with other nonphysiological constructs [44,78,79].

[*PSI*^+^] formation is a very rare event that can be induced by transient overexpression of Sup35p or that of its prion domain Sup35NM [18]. In both cases, however, [*PSI*^+^] formation de novo requires the presence of the prion-inducing [*PIN*^+^] prion, which corresponds to the prion form of Rnq1p, a protein of unclear function, although other aggregation-prone proteins such as polyglutamine-containing polypeptides can substitute for it [24,80,81,82]. The underlying mechanism is unclear: Rnq1p aggregates could act as “decoys” keeping molecular chaperones and other antiprion systems busy, or they could act as an “aggregation platform” that favors Sup35p conversion to a prion form [83]. Recombinant Sup35p fibrils are infectious, meaning that they trigger [*PSI*^+^] formation when introduced inside [*psi*^-^] recipient cells [84,85]. Importantly, [*PSI*^+^] induction using transformation of recombinant Sup35p fibrils bypasses the need for [*PIN*^+^] (this is also true when cell extracts from [*PSI*^+^] cells are used as infectious material [44,86,87]). Fibrils obtained under different experimental conditions reproducibly induce distinct [*PSI*^+^] strains in a concentration-dependent manner, suggesting they can directly recruit and template endogenous Sup35p monomers to generate self-replicating propagons [84,85]. However, given the extremely high conversion rates achieved in protein transformation assays [84,86,87,88], recombinant fibrils may also act as [*PIN*^+^]-like factors by sequestering critical antiprion determinants. Addressing this issue experimentally is difficult, as the protein transformation procedure relies on polyethylene glycol-induced fusion of spheroplasts that are subsequently regenerated and cultivated for ~20 generations to form colonies on selective plates. These colonies undergo a second round of replication on plates, allowing the scoring of [*psi*^-^] and [*PSI*^+^] colonies. Hence, the intracellular fate (e.g., chaperone-mediated fragmentation, clearance, specific subcellular localization) and immediate consequences (e.g., interactions with cellular factors, seeding of Sup35p aggregation) of the transforming material are eventually lost by dilution cell division after cell division [84,86,87,89]. Hence, it is difficult to conclude to which extent recombinant fibrils resemble propagons and behave as such when introduced within recipient cells.

Liebman and colleagues reported the purification of SDS-resistant particles from [*PSI*^+^] cells expressing hexahistidine-tagged Sup35p [56]. Using negative staining EM, they showed that these particles have the aspect of ~20 nm barrels composed of ~4–20 Sup35p molecules associated with molecular chaperones, mainly Hsp70-family members [56]. These barrel-like structures, which could be small amyloid fragments, often associated to form ~100–200 nm detergent-labile bundles [56]. Purified Sup35p aggregates induced [*PSI*^+^] in protein transformation assays; when treated with SDS, the infectivity increased, suggesting that the ~20 nm barrel-like Sup35p polymers are the infectious unit [56]. In agreement with these results, we showed that the size, amount and infectious properties of SDS-resistant Sup35p particles changes during growth and according to the metabolic status of the cells [44,47]. This could be due to growth phase-dependent changes in the association of Sup35p prion particles with molecular chaperones and/or in their organization in higher order aggregates, as suggested by fluorescence microscopy data [44].

**Figure 4:**
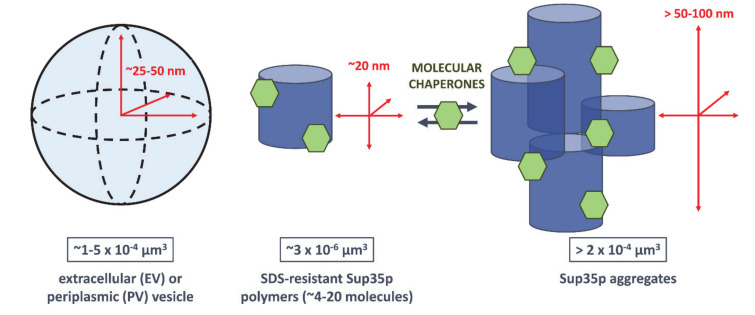
Average volumes of vesicles, detergent-resistant Sup35 polymers and larger Sup35p aggregates. The average volume of extracellular or periplasmic vesicles, considered for simplicity to be perfect spheres, was estimated based on the average diameter (~50–100 nm) of Sup35p-containing vesicles [6,45,47]. The volume of SDS-resistant Sup35p polymers and associated proteins (e.g., molecular chaperones, *green hexagons*) was calculated based on the ~20 nm barrel-like structures previously detected by electron microscopy [56]. The volume of the larger Sup35p aggregates was estimated by considering the association of three to four of these barrel-like structures [56].

We showed that Sup35p prion particles are exported to the extracellular medium via extracellular vesicles (EV) [45]. The size of these vesicle-encapsulated SDS-resistant Sup35p prion particles was the same as those in cell extracts from exponentially growing cells [45]. Importantly, the infectivity of cytosolic and EV-encapsulated Sup35p prion particles, assessed by protein transformation assays at comparable Sup35p levels, was the same [45]. Thus, we hypothesized that the infectious particles packaged within vesicles could be actual propagons [6,43]. For the sake of comparison, I here estimated the average volume of Sup35p-containing EV, which were ~50–100 nm in diameter, and that of the barrel-like SDS-resistant and larger SDS-labile Sup35p assemblies described by Liebman and colleagues [45,56] (Figure 4). It is evident that while EV could contain one or several SDS-resistant Sup35p particles, they are too small for larger aggregates [56] or fibrillar bundles [74] (Figure 4). We cannot however exclude that Sup35p prion particles undergo molecular rearrangements or condensation upon packaging within vesicles or that larger amyloid fragments stick to the vesicle surface or bind to peripheral vesicle proteins. Purifying Sup35p prion particles from vesicles and comparing them to those isolated from cytosolic extracts would be of great interest to characterize infectious Sup35p particles at the molecular level. Such an endeavor could be facilitated by using strains expressing hexahistidine-tagged Sup35p [56] and under growth conditions where large amounts of Sup35p-containing vesicles are easily extractable from the yeast periplasm [47].

## 4. Vesicle-Encapsulated Propagons May Overcome Spatial Quality Control during Bud Formation

According to our own observations and those reported in the literature, yeast prions exhibit a wide range of mitotic stabilities. Intrinsic properties of each prion variant, the genetic background of the yeast strains carrying the prions, as well as metabolic and environmental factors affect the transmission of propagons [18,19,53,68,69,70,90]. As discussed above, because of overlapping protein quality control, antiprion and antiaging systems, prion loss is expected to occur, albeit at low frequencies at each cell division, particularly in young cells [10,64,65,66]. However, the mitotic stabilities of several [*PSI*^+^], [*URE3*] or [*PIN*^+^] variants are on par with those of chromosomes or centromere plasmids: even after extended cultivation periods or hundreds of generations, prion-free cells or sectored colonies seldom appear. Whereas antiprion systems may be mostly efficient in ridding cells of harmful prion variants [10], neutral or beneficial prions may be poorer substrates for such systems. As discussed above, extracellular or periplasmic vesicles are physically able to contain and carry small SDS-resistant infectious prion particles (Figure 4) [45,47,56]. Using protein transformation assays, we showed that these vesicles were able to induce [*PSI*^+^] in [*psi*^-^, *pin*^-^] cells, indicating that they contain Sup35p propagons [45,47]. Prior to their export in the periplasm or the extracellular space, these vesicles may accumulate as IVC [51] and constitute a pool of “hidden” infectious Sup35p prion particles [45,47]. In light of these findings, we proposed a prion propagation model that takes into account their packaging within vesicles (Figure 2) [6,43]. We believe the very high mitotic stability of some prion variants could be accounted for by a vesicle-mediated mother-to-bud transmission [6,43]. Secretory vesicles deliver the macromolecules and enzymes that are required to build the plasma membrane and cell wall of the emerging yeast bud [61]. Small mobile prion-containing vesicles could join this flow of vesicles transported across the bud neck and deliver prion particles to daughter cells [43]. The polarized transport of vesicles, as well as organelles, from mother to daughter cells depends on actin cables (Figure 2) [91]. Remarkably, yeast prion formation was shown to depend upon components of the actin cortical cytoskeleton and the endocytic–vacuolar pathway [92,93,94,95]. Furthermore, the transport of prion amyloid aggregates to the insoluble protein deposit (IPOD) inclusion sites relies on an actin cable-based recruitment machinery that also involves vesicular transport [96]. Hence, prion-containing vesicles could join the flow of secretory vesicles trafficking to the bud, ensuring efficient and stable transmission of propagons at each cell division (Figure 2). Vesicles would serve as a cloak, protecting propagons from the action of molecular chaperones, proteolytic machineries and other spatial quality control and antiprion systems [43]. Periplasmic vesicles may avoid molecular crowding and “checkpoint” controls at the bud neck and simply diffuse to daughter cells where they could be reinternalized depending on growth conditions and the metabolic state of the cells (Figure 2) [43].

The existence of this vesicle-based prion transmission mechanism still needs further experimental proof, and some issues, in particular regarding prion curing, need to be addressed. Millimolar concentrations of guanidine hydrochloride, which inactivate the Hsp104 disaggregase, cure most yeast prions [97]. Hsp104, in cooperation with Hsp70 and Hsp40 family members (e.g., Ssa1p, Sis1p), plays an essential role in the propagation of most yeast prions by remodeling large aggregates into smaller species [23,41,98,99,100,101]. Hsp104 also plays key roles in asymmetric segregation of protein aggregates and has been shown to interact with endocytic vesicle-associated proteins involved in the sequestration of protein aggregates [102,103,104,105,106,107]. How can guanidine hydrochloride inactivation of Hsp104 rid cells of vesicle-encapsulated prions? [*PSI*^+^] curing takes at least four to six generations before the first [*psi*^-^] cells are detected in the population [54,108,109]. During this lag time, the number of propagons decreases to a point where daughter cells fail to receive any remaining prion particle [54]. Hsp104 inactivation causes a shift toward larger prion aggregates, which are in equilibrium with propagons [110]. As the number of cytosolic propagons decreases, their incorporation within vesicles should logically decrease as well. Furthermore, extracellular vesicles were shown to carry Hsp104, Sis1p and Ssa1p [45,51]. These molecular chaperones may engage and associate with propagons [56] within the vesicles, an activity that could be prevented by the guanidine hydrochloride treatment.

## 5. Concluding Remarks

Our recent reports suggest the cause-and-effect relationship between macroscopic hallmarks of [*PSI*^+^] (e.g., SDS-resistant Sup35p particles, fluorescent Sup35GFP foci) and [*PSI*^+^] propagons is not as straightforward as previously thought [44,47,53]. In this and previous reviews, we discussed how EV could help us to access the true physical nature of yeast prions’ transmissible entities or propagons [6,43]. Deciphering the mechanisms of spatiotemporal selection and packaging of prion particles inside vesicles is of great interest not only for the yeast prions field but also for human biology [6,43]. Different types of EV (e.g., exosomes, microvesicles, ectosomes) are able to secrete misfolded assemblies of pathological proteins, such as the prion protein PrP, α-synuclein or Tau and contribute to their prion-like spreading pattern in various neurodegenerative diseases (e.g., Creutzfeldt–Jakob, Parkinson’s, Alzheimer’s diseases) [111,112,113,114,115].

Fundamental cellular functions, including protein quality control, secretory pathway and endosomal–autophagic processes, are conserved from yeast to humans. Therefore, our recent work suggests yeasts could constitute a powerful model to investigate the mechanisms and molecular determinants that govern the selection, targeting and vesicle packaging of pathological prion-like protein aggregates in neurodegenerative diseases [43]. Heterologous expression of pathological human proteins in yeast allows the recapitulation of most of their aggregation and cytotoxic properties. Furthermore, known modifiers of protein aggregation, such as molecular chaperones or proteolytic systems, are conserved in yeast and are able to act on these prion proteins [43,116,117,118]. Yeast offers a vast array of fast and cost-effective experimental possibilities, including but not limited to the availability of genome-wide deletion and mutant libraries or automated high-throughput drug screening. While the isolation of EV from yeast culture supernatant is a cumbersome process poorly suited for screening purposes, the isolation of PV is by contrast quick, easy and compatible with high-throughput assays [47]. We predict that developing yeast models of vesicle-mediated prion proteins export will allow us not only to gain fundamental knowledge of the underlying mechanisms but also to design alternative strategies against the propagation of neuropathological protein assemblies.

## Figures and Tables

**Table 1:** Amyloid-based prions in the yeast *Saccharomyces cerevisiae* (adapted from [8,10,21]).

Prion	Protein	Protein Function	Prion Phenotypes	References
[*PSI*^+^]	Sup35p	translation terminator factor (eRF3)	reduced function results in increased nonsense suppression; impaired growth; growth advantage under stress conditions; increased chronological lifespan ^§^	[3,22,23]
[*URE3*]	Ure2p	nitrogen regulation (poor nitrogen sources repression)	loss of function; slow growth	[1,5]
[*PIN^+^*]	Rnq1p	unknown	induction of [*PSI*^+^] or [*URE3*] formation	[24]
[*OCT*^+^]	Cyc8p	transcription repression	impaired mating and sporulation	[25]
[*SWI*^+^]	Swi1p	chromatin remodeling; transcription regulation	poor growth on non-fermentable carbon sources	[26]
[*MOT3*^+^]	Mot3p	transcription regulation	acquisition of multicellular growth forms (biofilms)	[27]
[*MOD*^+^]	Mod5p	tRNA isopentenyltransferase	fluconazole resistance; slow growth	[28]
[*LSB*^+^]	Lsb2p	actin nucleation inhibition	thermal stress-induced [*PIN*^+^] activity	[29]

^§^ Depending on the prion strain and environmental or genetic conditions, some prion-associated phenotypes can de deleterious or advantageous to yeast [7,30,31,32,33,34,35,36]. Non amyloid-based epigenetic elements were also reported in yeast (e.g., [*BETA*], [*ISP*^+^], [*SMAUG*^+^], [*GAR*^+^] [37,38,39,40]).

## Data Availability

Not applicable.

## Abbreviations

EV	Extracellular vesicles
PV	Periplasmic vesicles
EM	Electron microscopy
VID	Vacuolar import and degradation
IVC	Intracellular vesicle clusters
IPOD	Insoluble protein deposit
JUNQ	Juxta nuclear quality control compartment

## References

[1] Aigle M., Lacroute F. (1975). Genetical aspects of [URE3], a non-mitochondrial, cytoplasmically inherited mutation in yeast. MGG Mol. Gen. Genet..

[2] Coustou V., Deleu C., Saupe S., Begueret J. (1997). The protein product of the het-s heterokaryon incompatibility gene of the fungus Podospora anserina behaves as a prion analog. Proc. Natl. Acad. Sci. USA.

[3] Cox B.S. (1965). PSI, a cytoplasmic suppressor of super-supressor in yeast. Heredity.

[4] Prusiner S.B. (1982). Novel proteinaceous infectious particles cause scrapie. Science.

[5] Wickner R.B. (1994). [URE3] as an altered URE2 protein: Evidence for a prion analog in Saccharomyces cerevisiae. Science.

[6] Kabani M., Melki R. (2016). More than just trash bins? Potential roles for extracellular vesicles in the vertical and horizontal transmission of yeast prions. Curr. Genet..

[7] Kabani M., Melki R. (2011). Yeast prions assembly and propagation: Contributions of the prion and non-prion moieties and the nature of assemblies. Prion.

[8] Liebman S.W., Chernoff Y.O. (2012). Prions in yeast. Genetics.

[9] Sindi S.S., Serio T.R. (2009). Prion dynamics and the quest for the genetic determinant in protein-only inheritance. Curr. Opin. Microbiol..

[10] Wickner R.B., Edskes H.K., Son M., Wu S., Niznikiewicz M. (2020). How Do Yeast Cells Contend with Prions?. Int. J. Mol. Sci..

[11] Glover J.R., Kowal A.S., Schirmer E.C., Patino M.M., Liu J.J., Lindquist S. (1997). Self-seeded fibers formed by Sup35, the protein determinant of [PSI+], a heritable prion-like factor of S. cerevisiae. Cell.

[12] Krzewska J., Melki R. (2006). Molecular chaperones and the assembly of the prion Sup35p, an in vitro study. EMBO J..

[13] Patel B.K., Liebman S.W. (2007). “Prion-proof” for [PIN+]: Infection with In Vitro-made Amyloid Aggregates of Rnq1p-(132-405) Induces [PIN+]. J. Mol. Biol..

[14] Thual C., Komar A.A., Bousset L., Fernandez-Bellot E., Cullin C., Melki R. (1999). Structural characterization of Saccharomyces cerevisiae prion-like protein Ure2. J. Biol. Chem..

[15] Taylor K.L., Cheng N., Williams R.W., Steven A.C., Wickner R.B. (1999). Prion domain initiation of amyloid formation in vitro from native Ure2p. Science.

[16] Bradley M.E., Edskes H.K., Hong J.Y., Wickner R.B., Liebman S.W. (2002). Interactions among prions and prion “strains” in yeast. Proc. Natl. Acad. Sci. USA.

[17] Bradley M.E., Liebman S.W. (2003). Destabilizing interactions among [PSI(+)] and [PIN(+)] yeast prion variants. Genetics.

[18] Derkatch I.L., Chernoff Y.O., Kushnirov V.V., Inge-Vechtomov S.G., Liebman S.W. (1996). Genesis and variability of [PSI] prion factors in Saccharomyces cerevisiae. Genetics.

[19] Brachmann A., Baxa U., Wickner R.B. (2005). Prion generation in vitro: Amyloid of Ure2p is infectious. EMBO J..

[20] Winkler J., Tyedmers J., Bukau B., Mogk A. (2012). Chaperone networks in protein disaggregation and prion propagation. J. Struct. Biol..

[21] Oamen H.P., Lau Y., Caudron F. (2020). Prion-like proteins as epigenetic devices of stress adaptation. Exp. Cell Res..

[22] Tuite M.F., Cox B.S. (2006). The [PSI+] prion of yeast: A problem of inheritance. Methods.

[23] Patino M.M., Liu J.J., Glover J.R., Lindquist S. (1996). Support for the prion hypothesis for inheritance of a phenotypic trait in yeast. Science.

[24] Derkatch I.L., Bradley M.E., Hong J.Y., Liebman S.W. (2001). Prions affect the appearance of other prions: The story of [PIN+]. Cell.

[25] Patel B.K., Gavin-Smyth J., Liebman S.W. (2009). The yeast global transcriptional co-repressor protein Cyc8 can propagate as a prion. Nat. Cell Biol..

[26] Du Z., Park K.W., Yu H., Fan Q., Li L. (2008). Newly identified prion linked to the chromatin-remodeling factor Swi1 in Saccharomyces cerevisiae. Nat. Genet..

[27] Alberti S., Halfmann R., King O., Kapila A., Lindquist S. (2009). A systematic survey identifies prions and illuminates sequence features of prionogenic proteins. Cell.

[28] Suzuki G., Shimazu N., Tanaka M. (2012). A yeast prion, Mod5, promotes acquired drug resistance and cell survival under environmental stress. Science.

[29] Chernova T.A., Kiktev D.A., Romanyuk A.V., Shanks J.R., Laur O., Ali M., Ghosh A., Kim D., Yang Z., Mang M. (2017). Yeast Short-Lived Actin-Associated Protein Forms a Metastable Prion in Response to Thermal Stress. Cell Rep..

[30] Wickner R.B., Edskes H.K., Bateman D., Kelly A.C., Gorkovskiy A. (2011). The yeast prions [PSI+] and [URE3] are molecular degenerative diseases. Prion.

[31] Tuite M.F. (2015). Yeast prions: Paramutation at the protein level?. Semin. Cell Dev. Biol..

[32] Wickner R.B., Shewmaker F.P., Bateman D.A., Edskes H.K., Gorkovskiy A., Dayani Y., Bezsonov E.E. (2015). Yeast prions: Structure, biology, and prion-handling systems. Microbiol. Mol. Biol. Rev..

[33] Garcia D.M., Jarosz D.F. (2014). Rebels with a cause: Molecular features and physiological consequences of yeast prions. FEMS Yeast Res..

[34] Halfmann R., Alberti S., Lindquist S. (2010). Prions, protein homeostasis, and phenotypic diversity. Trends Cell Biol..

[35] Newby G.A., Lindquist S. (2013). Blessings in disguise: Biological benefits of prion-like mechanisms. Trends Cell Biol..

[36] Shorter J., Lindquist S. (2005). Prions as adaptive conduits of memory and inheritance. Nat. Rev. Genet..

[37] Roberts B.T., Wickner R.B. (2003). Heritable activity: A prion that propagates by covalent autoactivation. Genes Dev..

[38] Rogoza T., Goginashvili A., Rodionova S., Ivanov M., Viktorovskaya O., Rubel A., Volkov K., Mironova L. (2010). Non-Mendelian determinant [ISP+] in yeast is a nuclear-residing prion form of the global transcriptional regulator Sfp1. Proc. Natl. Acad. Sci. USA.

[39] Jarosz D.F., Lancaster A.K., Brown J.C., Lindquist S. (2014). An evolutionarily conserved prion-like element converts wild fungi from metabolic specialists to generalists. Cell.

[40] Chakravarty A.K., Smejkal T., Itakura A.K., Garcia D.M., Jarosz D.F. (2020). A Non-amyloid Prion Particle that Activates a Heritable Gene Expression Program. Mol. Cell.

[41] Chernoff Y.O., Lindquist S.L., Ono B., Inge-Vechtomov S.G., Liebman S.W. (1995). Role of the chaperone protein Hsp104 in propagation of the yeast prion-like factor [psi+]. Science.

[42] Cox B., Ness F., Tuite M. (2003). Analysis of the generation and segregation of propagons: Entities that propagate the [PSI+] prion in yeast. Genetics.

[43] Kabani M. (2020). Hiding in plain sight: Vesicle-mediated export and transmission of prion-like proteins. Microb. Cell.

[44] Wang K., Melki R., Kabani M. (2019). Growth phase-dependent changes in the size and infectivity of SDS-resistant Sup35p assemblies associated with the [PSI+] prion in yeast. Mol. Microbiol..

[45] Kabani M., Melki R. (2015). Sup35p in its soluble and prion states is packaged inside extracellular vesicles. MBio.

[46] Rodrigues M.L., Casadevall A. (2018). A two-way road: Novel roles for fungal extracellular vesicles. Mol. Microbiol..

[47] Kabani M., Pilard M., Melki R. (2020). Glucose availability dictates the export of the soluble and prion forms of Sup35p via periplasmic or extracellular vesicles. Mol. Microbiol..

[48] Huang P.H., Chiang H.L. (1997). Identification of novel vesicles in the cytosol to vacuole protein degradation pathway. J. Cell Biol..

[49] Giardina B.J., Stein K., Chiang H.-L. (2014). The endocytosis gene END3 is essential for the glucose-induced rapid decline of small vesicles in the extracellular fraction in Saccharomyces cerevisiae. J. Extracell. Vesicles.

[50] Giardina B.J., Stanley B.A., Chiang H.L. (2014). Glucose induces rapid changes in the secretome of Saccharomyces cerevisiae. Proteome Sci..

[51] Winters C.M., Hong-Brown L.Q., Chiang H.L. (2020). Intracellular vesicle clusters are organelles that synthesize extracellular vesicle-associated cargo proteins in yeast. J. Biol. Chem..

[52] Derdowski A., Sindi S.S., Klaips C.L., DiSalvo S., Serio T.R. (2010). A Size Threshold Limits Prion Transmission and Establishes Phenotypic Diversity. Science.

[53] Wang K., Melki R., Kabani M. (2017). A prolonged chronological lifespan is an unexpected benefit of the [PSI+] prion in yeast. PLoS ONE.

[54] Byrne L.J., Cole D.J., Cox B.S., Ridout M.S., Morgan B.J.T., Tuite M.F. (2009). The Number and Transmission of [PSI+] Prion Seeds (Propagons) in the Yeast Saccharomyces cerevisiae. PLoS ONE.

[55] Speldewinde S.H., Grant C.M. (2017). The frequency of yeast [PSI+] prion formation is increased during chronological ageing. Microb. Cell.

[56] Bagriantsev S.N., Gracheva E.O., Richmond J.E., Liebman S.W. (2008). Variant-specific [PSI+] Infection Is Transmitted by Sup35 Polymers within [PSI+] Aggregates with Heterogeneous Protein Composition. Mol. Biol. Cell.

[57] Villali J., Dark J., Brechtel T.M., Pei F., Sindi S.S., Serio T.R. (2020). Nucleation seed size determines amyloid clearance and establishes a barrier to prion appearance in yeast. Nat. Struct. Mol. Biol..

[58] Tanaka M., Collins S.R., Toyama B.H., Weissman J.S. (2006). The physical basis of how prion conformations determine strain phenotypes. Nature.

[59] Juanes M.A., Piatti S. (2016). The final cut: Cell polarity meets cytokinesis at the bud neck in S. cerevisiae. Cell. Mol. Life Sci..

[60] Bhavsar-Jog Y.P., Bi E. (2017). Mechanics and regulation of cytokinesis in budding yeast. Semin. Cell Dev. Biol..

[61] Sentandreu R., Northcote D.H. (1969). The formation of buds in yeast. J. Gen. Microbiol..

[62] Knoblach B., Rachubinski R.A. (2015). Sharing the cell’s bounty—Organelle inheritance in yeast. J. Cell Sci..

[63] Higuchi-Sanabria R., Pernice W.M.A., Vevea J.D., Alessi Wolken D.M., Boldogh I.R., Pon L.A. (2014). Role of asymmetric cell division in lifespan control in Saccharomyces cerevisiae. FEMS Yeast Res..

[64] Sontag E.M., Samant R.S., Frydman J. (2017). Mechanisms and Functions of Spatial Protein Quality Control. Annu. Rev. Biochem..

[65] Hill S.M., Hanzén S., Nyström T. (2017). Restricted access: Spatial sequestration of damaged proteins during stress and aging. EMBO Rep..

[66] Chernova T.A., Wilkinson K.D., Chernoff Y.O. (2017). Prions, chaperones, and proteostasis in yeast. Cold Spring Harb. Perspect. Biol..

[67] Kalastavadi T., True H.L. (2010). Analysis of the [RNQ+] prion reveals stability of amyloid fibers as the key determinant of yeast prion variant propagation. J. Biol. Chem..

[68] Huang V.J., Stein K.C., True H.L. (2013). Spontaneous Variants of the [RNQ+] Prion in Yeast Demonstrate the Extensive Conformational Diversity Possible with Prion Proteins. PLoS ONE.

[69] McGlinchey R.P., Kryndushkin D., Wickner R.B. (2011). Suicidal [PSI+] is a lethal yeast prion. Proc. Natl. Acad. Sci. USA.

[70] Schlumpberger M., Prusiner S.B., Herskowitz I. (2001). Induction of distinct [URE3] yeast prion strains. Mol. Cell. Biol..

[71] Krzewska J., Tanaka M., Burston S.G., Melki R. (2007). Biochemical and functional analysis of the assembly of full-length Sup35p and its prion-forming domain. J. Biol. Chem..

[72] Schutz A.K., Habenstein B., Luckgei N., Bousset L., Sourigues Y., Nielsen A.B., Melki R., Bockmann A., Meier B.H. (2014). Solid-state NMR sequential assignments of the amyloid core of full-length Sup35p. Biomol. NMR Assign..

[73] Baxa U., Keller P.W., Cheng N., Wall J.S., Steven A.C. (2011). In Sup35p filaments (the [PSI+] prion), the globular C-terminal domains are widely offset from the amyloid fibril backbone. Mol. Microbiol..

[74] Saibil H.R., Seybert A., Habermann A., Winkler J., Eltsov M., Perkovic M., Castano-Diez D., Scheffer M.P., Haselmann U., Chlanda P. (2012). Heritable yeast prions have a highly organized three-dimensional architecture with interfiber structures. Proc. Natl. Acad. Sci. USA.

[75] Arslan F., Hong J.Y., Kanneganti V., Park S.-K., Liebman S.W. (2015). Heterologous Aggregates Promote De Novo Prion Appearance via More than One Mechanism. PLoS Genet..

[76] Mathur V., Taneja V., Sun Y., Liebman S.W. (2010). Analyzing the Birth and Propagation of Two Distinct Prions, [PSI+] and [Het-s]y, in Yeast. Mol. Biol. Cell.

[77] Sharma J., Wisniewski B.T., Paulson E., Obaoye J.O., Merrill S.J., Manogaran A.L. (2017). De novo [PSI (+)] prion formation involves multiple pathways to form infectious oligomers. Sci. Rep..

[78] Song Y., Wu Y.X., Jung G., Tutar Y., Eisenberg E., Greene L.E., Masison D.C. (2005). Role for Hsp70 chaperone in Saccharomyces cerevisiae prion seed replication. Eukaryot. Cell.

[79] Satpute-Krishnan P., Serio T.R. (2005). Prion protein remodelling confers an immediate phenotypic switch. Nature.

[80] Derkatch I.L., Bradley M.E., Zhou P., Chernoff Y.O., Liebman S.W. (1997). Genetic and environmental factors affecting the de novo appearance of the [PSI+] prion in Saccharomyces cerevisiae. Genetics.

[81] Derkatch I.L., Uptain S.M., Outeiro T.F., Krishnan R., Lindquist S.L., Liebman S.W. (2004). Effects of Q/N-rich, polyQ, and non-polyQ amyloids on the de novo formation of the [PSI+] prion in yeast and aggregation of Sup35 in vitro. Proc. Natl. Acad. Sci. USA.

[82] Bui Q., Sherma J., Hines J.K. (2018). Using high performance thin layer chromatography-densitometry to study the influence of the prion [RNQ+] and its determinant prion protein Rnq1 on yeast lipid profiles. Separations.

[83] Serio T.R. (2018). [PIN + ]ing down the mechanism of prion appearance. FEMS Yeast Res..

[84] Tanaka M., Chien P., Naber N., Cooke R., Weissman J.S. (2004). Conformational variations in an infectious protein determine prion strain differences. Nature.

[85] King C.Y., Diaz-Avalos R. (2004). Protein-only transmission of three yeast prion strains. Nature.

[86] Kabani M., Cosnier B., Bousset L., Rousset J.P., Melki R., Fabret C. (2011). A mutation within the C-terminal domain of Sup35p that affects [PSI+] prion propagation. Mol. Microbiol..

[87] Kabani M., Redeker V., Melki R. (2014). A role for the proteasome in the turnover of sup35p and in [PSI+] prion propagation. Mol. Microbiol..

[88] Bousset L., Luckgei N., Kabani M., Gardiennet C., Schütz A.K., Melki R., Meier B.H., Böckmann A. (2020). Prion Amyloid Polymorphs—The Tag Might Change It All. Front. Mol. Biosci..

[89] Tanaka M., Weissman J.S. (2006). An Efficient Protein Transformation Protocol for Introducing Prions into Yeast. Methods Enzymol..

[90] Sharma D., Martineau C.N., Le Dall M.T., Reidy M., Masison D.C., Kabani M. (2009). Function of SSA subfamily of Hsp70 within and across species varies widely in complementing Saccharomyces cerevisiae cell growth and prion propagation. PLoS ONE.

[91] Pruyne D., Legesse-Miller A., Gao L., Dong Y., Bretscher A. (2004). Mechanisms of polarized growth and organelle segregation in yeast. Annu. Rev. Cell Dev. Biol..

[92] Ganusova E.E., Ozolins L.N., Bhagat S., Newnam G.P., Wegrzyn R.D., Sherman M.Y., Chernoff Y.O. (2006). Modulation of prion formation, aggregation, and toxicity by the actin cytoskeleton in yeast. Mol. Cell. Biol..

[93] Manogaran A.L., Hong J.Y., Hufana J., Tyedmers J., Lindquist S., Liebman S.W. (2011). Prion formation and polyglutamine aggregation are controlled by two classes of genes. PLoS Genet..

[94] Speldewinde S.H., Doronina V.A., Tuite M.F., Grant C.M. (2017). Disrupting the cortical actin cytoskeleton points to two distinct mechanisms of yeast [PSI+] prion formation. PLoS Genet..

[95] Dorweiler J.E., Oddo M.J., Lyke D.R., Reilly J.A., Wisniewski B.T., Davis E.E., Kuborn A.M., Merrill S.J., Manogaran A.L. (2020). The actin cytoskeletal network plays a role in yeast prion transmission and contributes to prion stability. Mol. Microbiol..

[96] Kumar R., Nawroth P.P., Tyedmers J. (2016). Prion Aggregates Are Recruited to the Insoluble Protein Deposit (IPOD) via Myosin 2-Based Vesicular Transport. PLoS Genet..

[97] Ferreira P.C., Ness F., Edwards S.R., Cox B.S., Tuite M.F. (2001). The elimination of the yeast [PSI+] prion by guanidine hydrochloride is the result of Hsp104 inactivation. Mol. Microbiol..

[98] Allen K.D., Wegrzyn R.D., Chernova T.A., Muller S., Newnam G.P., Winslett P.A., Wittich K.B., Wilkinson K.D., Chernoff Y.O. (2005). Hsp70 chaperones as modulators of prion life cycle: Novel effects of Ssa and Ssb on the Saccharomyces cerevisiae prion [PSI+]. Genetics.

[99] Tipton K.A., Verges K.J., Weissman J.S. (2008). In Vivo Monitoring of the Prion Replication Cycle Reveals a Critical Role for Sis1 in Delivering Substrates to Hsp104. Mol. Cell.

[100] Sondheimer N., Lopez N., Craig E.A., Lindquist S. (2001). The role of Sis1 in the maintenance of the [RNQ+] prion. EMBO J..

[101] Glover J.R., Lindquist S. (1998). Hsp104, Hsp70, and Hsp40: A novel chaperone system that rescues previously aggregated proteins. Cell.

[102] Zhou C., Slaughter B.D., Unruh J.R., Eldakak A., Rubinstein B., Li R. (2011). Motility and segregation of Hsp104-associated protein aggregates in budding yeast. Cell.

[103] Tessarz P., Schwarz M., Mogk A., Bukau B. (2009). The yeast AAA+ chaperone Hsp104 is part of a network that links the actin cytoskeleton with the inheritance of damaged proteins. Mol. Cell. Biol..

[104] Erjavec N., Larsson L., Grantham J., Nyström T. (2007). Accelerated aging and failure to segregate damaged proteins in Sir2 mutants can be suppressed by overproducing the protein aggregation-remodeling factor Hsp104p. Genes Dev..

[105] Liu B., Larsson L., Caballero A., Hao X., Öling D., Grantham J., Nyström T. (2010). The Polarisome Is Required for Segregation and Retrograde Transport of Protein Aggregates. Cell.

[106] Liu B., Larsson L., Franssens V., Hao X., Hill S.M., Andersson V., Hoglund D., Song J., Yang X., Oling D. (2011). Segregation of protein aggregates involves actin and the polarity machinery. Cell.

[107] Hill S.M., Hao X., Grönvall J., Spikings-Nordby S., Widlund P.O., Amen T., Jörhov A., Josefson R., Kaganovich D., Liu B. (2016). Asymmetric Inheritance of Aggregated Proteins and Age Reset in Yeast Are Regulated by Vac17-Dependent Vacuolar Functions. Cell Rep..

[108] Byrne L.J., Cox B.S., Cole D.J., Ridout M.S., Morgan B.J.T., Tuite M.F. (2007). Cell division is essential for elimination of the yeast [PSI+] prion by guanidine hydrochloride. Proc. Natl. Acad. Sci. USA.

[109] Eaglestone S.S., Ruddock L.W., Cox B.S., Tuite M.F. (2000). Guanidine hydrochloride blocks a critical step in the propagation of the prion-like determinant [PSI(+)] of Saccharomyces cerevisiae. Proc. Natl. Acad. Sci. USA.

[110] Kryndushkin D.S., Alexandrov I.M., Ter-Avanesyan M.D., Kushnirov V. (2003). V Yeast [PSI+] prion aggregates are formed by small Sup35 polymers fragmented by Hsp104. J. Biol. Chem..

[111] Fevrier B., Vilette D., Archer F., Loew D., Faigle W., Vidal M., Laude H., Raposo G. (2004). Cells release prions in association with exosomes. Proc. Natl. Acad. Sci. USA.

[112] Dujardin S., Bégard S., Caillierez R., Lachaud C., Delattre L., Carrier S., Loyens A., Galas M.C., Bousset L., Melki R. (2014). Ectosomes: A new mechanism for non-exosomal secretion of Tau protein. PLoS ONE.

[113] Rajendran L., Bali J., Barr M.M., Court F.A., Kramer-Albers E.-M., Picou F., Raposo G., van der Vos K.E., van Niel G., Wang J. (2014). Emerging Roles of Extracellular Vesicles in the Nervous System. J. Neurosci..

[114] Alvarez-Erviti L., Seow Y., Schapira A.H., Gardiner C., Sargent I.L., Wood M.J.A., Cooper J.M. (2011). Lysosomal dysfunction increases exosome-mediated alpha-synuclein release and transmission. Neurobiol. Dis..

[115] Minakaki G., Menges S., Kittel A., Emmanouilidou E., Schaeffner I., Barkovits K., Bergmann A., Rockenstein E., Adame A., Marxreiter F. (2018). Autophagy inhibition promotes SNCA/alpha-synuclein release and transfer via extracellular vesicles with a hybrid autophagosome-exosome-like phenotype. Autophagy.

[116] Dhakal S., Macreadie I. (2020). Protein homeostasis networks and the use of yeast to guide interventions in alzheimer’s disease. Int. J. Mol. Sci..

[117] Ishikawa T. (2020). Saccharomyces cerevisiae in neuroscience: How unicellular organism helps to better understand prion protein?. Neural Regen. Res..

[118] Tuite M.F. (2019). Yeast models of neurodegenerative diseases. Progress in Molecular Biology and Translational Science.

